# Demographic risk factors for mid-urethral sling failure. Do they really matter?

**DOI:** 10.1371/journal.pone.0207185

**Published:** 2018-11-12

**Authors:** Wojciech Majkusiak, Andrzej Pomian, Edyta Horosz, Aneta Zwierzchowska, Paweł Tomasik, Wojciech Lisik, Ewa Barcz

**Affiliations:** 1 Multidisciplinary Hospital Warsaw-Miedzylesie, Department of Obstetrics and Gynecology, Warsaw, Poland; 2 First Department of Obstetrics and Gynecology, Medical University of Warsaw, Warsaw, Poland; 3 Department of General Surgery and Transplantology, Medical University of Warsaw, Warsaw, Poland; University of Alberta, CANADA

## Abstract

Age, obesity and vaginal deliveries (VD) are recognized risk factors for stress urinary incontinence (SUI). According to many authors, the abovementioned risk factors for incontinence also increase the risk of mid-urethral sling (MUS) failure. Our aim was to evaluate the objective and subjective effectiveness of retropubic MUS in 12 months observation, relative to the three potential risk factors of failure: obesity, age and VDs. A prospective observational study including 238 women who underwent retropubic MUS implantation was performed. Patients were divided into subgroups: obese vs non-obese, <65 vs ≥65 years old and no history of VD vs ≥1 VD. Follow-up took place between 6 and 12 months post-surgery. Cough test, 1-hour pad test, pelvic floor ultrasound examination, and Incontinence Impact Questionnaire 7 (IIQ-7) results were assessed pre- and post-operatively. Of the 238 patients, 208 (86.3%) completed a minimum follow-up period of 12 months. **S**ignificant improvement in the pad test was observed in all patients (83.2 ± 78.6 g vs 0.7 ± 3.3 g). Negative cough test results were obtained in over 94% of patients. Significant improvement in the IIQ7 results was observed in all patients (74.2 ± 17.7 vs 5.5 ± 13.4). No significant differences in all the analyzed parameters with regard to BMI, age and parity were observed. No combination of risk factors influenced the objective and subjective cure rates. Our study demonstrated that older age, obesity and history of VDs have no impact on objective and subjective sling effectiveness in a short term observation. There is no influence of combined demographic features on the failure risk.

## Introduction

Urinary incontinence is a highly prevalent problem affecting over 40% of adult women [1].

Stress urinary incontinence (SUI), the most common incontinence type, influences the individual’s health related quality of life. Moreover, it is an economic problem connected with high personal and social costs. The incidence of incontinence increases with age and in elderly patients reaches over 50% [2].

Obesity is an important risk factor for SUI. The incidence of incontinence among women with body mass index (BMI) over 35 reaches 67.3% [3]. Other factors associated with higher incontinence rates are pregnancies and vaginal deliveries. Here, the negative impact of increased birthweight and maternal weight gain, as well as instrumental delivery is emphasized [4,5].

Mid-urethral slings (MUS) are the gold standard for surgical treatment of SUI. They seem to be an efficient and safe option for the affected patients. Recent meta-analysis evaluating the effectiveness of sub-urethral slings showed that in short-term observation the rates of subjective cure of transobturator (TOT- transobturator tape) and retropubic (TVT- tension-free vaginal tape) slings are similar (RR 0.98, 95% CI 0.96 to 1.00), ranging from 62% to 98% in the TOT group, and from 71% to 97% in the TVT group. Also, short-term objective cure rates were alike in both groups (RR 0.98, 95% CI 0.96 to 1.00) [6].

However, despite the relatively high cure rate there still remains a group of patients who do not benefit from the surgery. In the current literature, there are conflicting data concerning risk factors for surgery failure. According to many authors, the risk factors for incontinence such as older age, increased parity and obesity are at the same time risk factors for surgery failure [7,8]. On the other hand, growing evidence suggests that sling failure is connected rather to surgery technique than to patients’ characteristics. It has been demonstrated that one of the most important factors influencing mid-urethral sling success rate is the appropriate sling location beneath the distal part of urethra (too proximal position relative to the bladder neck is connected with persistent urinary incontinence) [9, 10, 11].

The aim of the present study was to evaluate the objective and subjective effectiveness of the retropubic MUS in short-term observation, taking into account the main three potential demographic risk factors of failure: obesity, age and parity as well as combinations of these three parameters.

## Materials and methods

The ethics committee of Medical University of Warsaw approved the study. It conforms to the Declaration of Helsinki. Written informed consent was obtained from all women during the preoperative consultation, concerning all medical interventions and the potential use of clinical data in future studies. Publication has been prepared in accordance with the STROBE checklist.

A prospective, observational, comparative study of all women who underwent primary incontinence surgery using the bottom–top retropubic MUS (Gynecare TVT blue, Ethicon, USA) was performed, between January 2013 and December 2016, at a tertiary University Hospital. The procedures were performed by 5 trained specialists.

In all cases, stress urinary incontinence was confirmed on the basis of medical history, and cough test in semi-sitting position with bladder filling approximately 300 mL (assessed with ultrasound examination). Cases of previous failed urinary incontinence surgery and concomitant pelvic organ prolapse (POPQ stage> 1) on pelvic examination were excluded. In none of the cases, pelvic floor reconstructive procedures were performed at the time of the surgery for SUI.

Patients were stratified to the following groups: obese (BMI≥ 30 kg/m2) vs non-obese (< 30 kg/m2) (according to the recommendation of the World Health Organization for the western population [12]. Analysis of surgery effectiveness was also performed in relation to the age (patients <65 vs ≥65 years old) and parity (multiparas vs patients with a history of 1 or more vaginal deliveries). Pre- and post-operative evaluation included the medical history, pelvic examination with a POPQ scale assessment, cough test, 1-hour pad test, urinalysis, and pelvic floor ultrasound examination. Urodynamic testing was performed only in cases of mixed urinary incontinence and subjects with detrusor over-activity were not scheduled for the surgery.

Follow-up was scheduled for 6 to 12 months post-surgery.

The quality of life was assessed pre- and postoperatively using the IIQ7 test (Incontinence Impact Questionnaire 7). In all cases, pelvic floor ultrasound examination was performed both before surgery and during the follow-up visit, according to Interdisciplinary S2k Guideline: Sonography in Urogynecology [13]. Examination was carried out in a standardized manner, with the patient in a semi-sitting position with the bladder filled to 300 mL, as previously described [14].

During the examination, bladder filling, residual post void volume as well as the individual urethral length was assessed before the surgery. Sling location in relation to the individual urethral length (%) measured as the distance from the bladder neck to the middle part of the sling.

The placement of the vaginal incision was adapted to the urethral length according to the one-third rule [15]. The sonographic length of the urethra was measured. Subsequently, the result was divided by three and thus the exact site of vaginal incision was defined.

The primary outcome of the study was the objective cure, defined as no urine leakage during the cough test performed with full bladder (approximately 300 ml) and negative 1-hour pad test (≤2 g). The secondary outcome was the surgery impact on quality of life assessed in IIQ7. We also performed analysis of intra- and postoperative complications such as: vaginal wall perforation, urinary bladder perforation, urinary retention (post-voiding residual volume > 100 ml), de novo overactive bladder (OAB), vaginal mucosal erosion and others. All the failures were analyzed individually. Location of the sling (in relation to individual urethral length) was assessed 6–12 months after the surgery.

Descriptive statistical analysis and statistical tests were performed using the R version 3.4.0 (by the R Foundation for Statistical Computing). Normality was tested using Lilliefors and Shapiro-Wilk W tests. We associated the degree and type of non-adherence using the Wilcoxon signed-rank and U Mann Whitney tests. For categorical data, chi-square and Fisher’s exact tests were used. Multiple regression was used for multivariable analysis. A post hoc sensitivity power analysis was conducted using the software package GPower (version 3.1.9.2).

## Results

238 patients fulfilled inclusion criteria and were included in the study. Demographic features of the examined group are summarized in Table 1.

**Table 1:** Demographic characteristics of the patients.

Patients	Total (n = 248)	Obese (n = 63)	Non-obese (n = 185)	Below 65 y.o. (n = 183)	Over 65 y.o. (n = 65)	VD = 0 (n = 17)	≥1 VD (n = 231)
Age (years)	57.5±10.4	58.7±10.6	56.8±10.1	52.9±7.3	70.8±5.1	60.4±9.1	57.3±10.4
BMI (kg/m^2^)	27.5±4.2	33.0±2.5	25.5±2.5	27.2 ±4.1	28.5 ±4.3	25.3 ±4.2	27.6 ±4.1
VD (no)	1.9±0.9	2.0 ±1.0	2.1±0.8	1.9±0.9	2.0 ±1.1	0	2.1 ±0.8
Urethral length (mm)	28.8 ±3.7	28.8 ±3.6	28.8 ±3.7	28.9 ±3.7	28.5 ±3.8	28.8±6.4	28.8 ±3.5

Data given as mean ± SD; VD–vaginal delivery; BMI- body mass index; y.o.- years old

In all cases, regardless of the demographic factors, distal sling location was accomplished (65.3+/-9.2% of urethral length).

10 patients underwent sling removal before final evaluation. In 6 cases, the sling was removed during the first 72 hours after the procedure due to bladder outlet obstruction and urinary retention. In 4 cases, sling removal took place 1–3 months post-surgery (1 because of urinary retention, 1 because of periurethral abscess, and in 2 cases due to urinary infections and overactive bladder syndrome occurring *de novo*). None of the sling was removed because of persistent or recurrent SUI.

206 out of 238 women (86.3%) underwent evaluation 6–12 months after the surgery. Mean length of follow up was 10.2 months.

In the examined group, 2 bladder perforations were diagnosed during the surgery and the catheterization was continued for 7 days after the procedure.

None of the patients was discharged with the Foley catheter or post-void residual volume >100 ml. We did not observe overactive bladder syndrome occurring *de novo* after the sling implantation. In none of the patients, vaginal erosion of the sling was observed.

Mean location of the tape in the cohort group was 66.2 ±87.3% of urethral length. None of them was located out of distal part of urethra.

### Objective cure rates

Statistically significant improvement in the 1-hour pad test was observed in all patients (Table 2).

**Table 2:** Mean results of the 1-hour pad test before and after the surgery in the compared subgroups of patients.

	Total	Obese	Non-obese	Below 65 y.o.	Over 65 y.o.	VD = 0	More than 1 VD
Before surgery	83.2 ±78.6 (n = 248)	76.1 ±72.6 (n = 63)	86.0 ±81.1 (n = 185)	83.9 ±81.6 (n = 183)	81.2 ±69.8 (n = 65)	56.1 ±37.4 (n = 17)	84.5 ±79.9 (n = 231)
6–12 months post-surgery	0.7 ±3.3 (n = 206)	1.2 ±5.5 (n = 51)	0.5 ±2.2 (n = 155)	0.5 ±2.3 (n = 152)	1.3 ±5.2 (n = 54)	0.3 ±0.8 (n = 15)	0.7 ±3.4 (n = 191)
Sensitivity*		0.41		0.40		0.68	
p-value**	<0.001	<0.001	<0.001	<0.001	<0.001	<0.001	<0.001

Data given as mean ± SD; VD–vaginal delivery; y.o.- years old

* 1- betta = 0.8; alfa = 0.05

** Wilcoxon signed-rank Test

**Fig 1:**
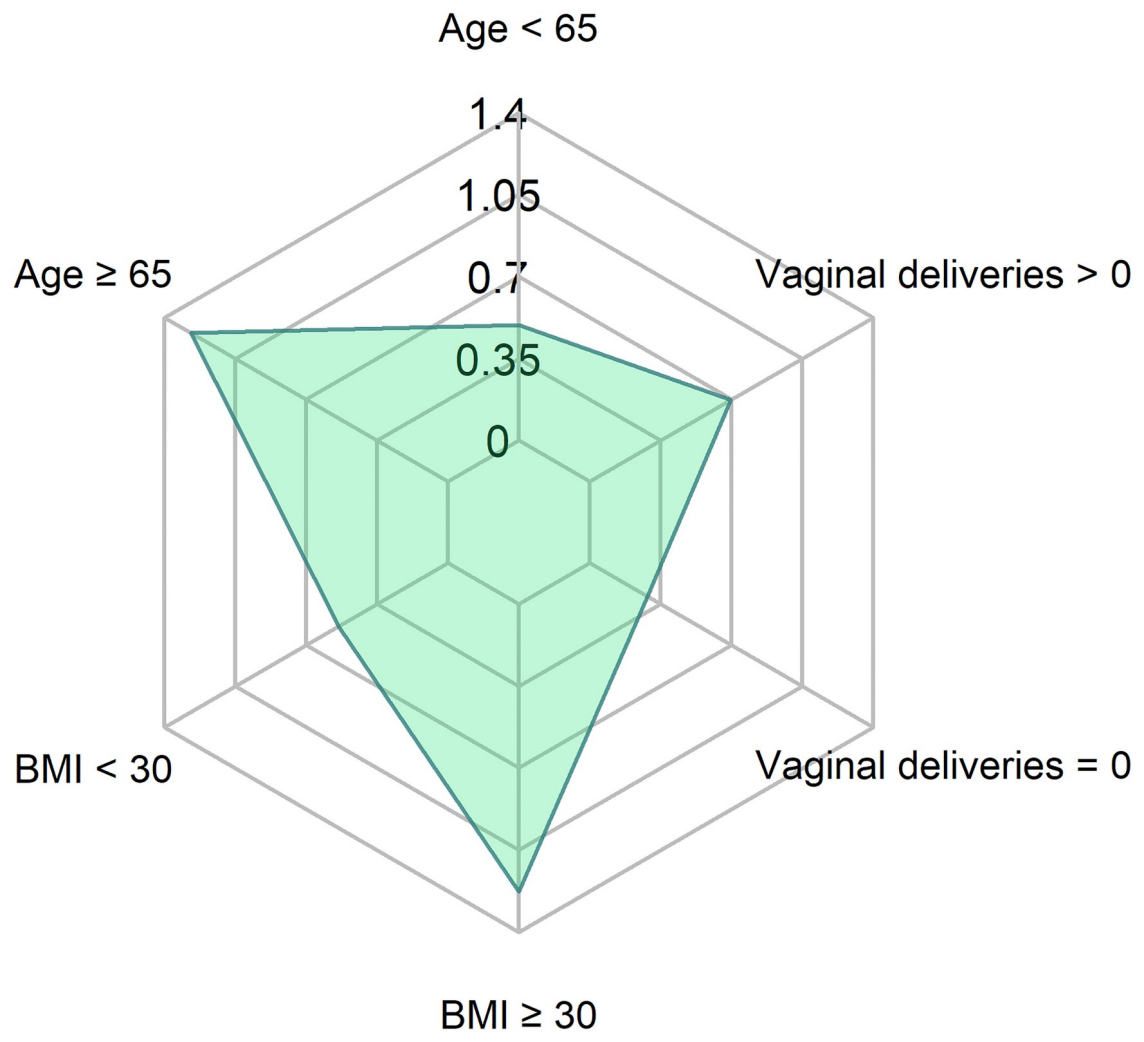
Mean 1 h-pad test results 6–12 months after surgery. p-values: obese vs non-obese: 0.79; <65 y.o. vs ≥ 65 y.o.: 0.51); VD = 0 vs ≥ 1 VD: 0.59 (U Mann Whitney Test). VD–vaginal delivery. BMI- body mass index (kg/m^2^).

No differences were observed in the improvement of 1-hour pad test between the subgroups as far as the demographic features are concerned (Fig 1).

In the whole group, negative cough test results were observed in over 94% of patients, and no significant differences depending on BMI, age and parity were observed (Table 3).

**Table 3:** Objective cure rate 6–12 months after surgery expressed as percent of negative cough test.

Negative cough test and 1-hour pad test						
Total (n = 194)	Obese (n = 47)	Non-obese (n = 147)	Below 65 y.o. (n = 144)	Over 65 y.o. (n = 50)	VD = 0 (n = 14)	More than 1 VD (n = 180)
94.2%	92.2%	94.8%	94.7%	92.6%	93.3%	94.2%
Sensitivity*	0.201					
p-value**	0.48		0.51		0.60	

VD–vaginal delivery; y.o.- years old

* 1- betta = 0.8; alfa = 0.05

** Fisher’s Exact Test

### Subjective cure rates

Significant improvement in the quality of life, measured as the difference in the result of the IIQ7 questionnaire assessed pre- and postoperatively, was observed in all the studied patients as well as in each subgroup (Table 4).

**Table 4:** The differences in the results of the IIQ7 questionnaire before and after surgery. Data given as mean ± SD.

	IIQ-7 results						
	Total	Obese	Non-obese	<65 y.o.	≥65 y.o	VD = 0	≥1 VD
Before surgery	74.2 ±17.7 (n = 248)	76.3 ±18.0 (n = 63)	73.8 ±16.8 (n = 185)	76.2 ±15.4 (n = 183)	68.4 ±22.5 (n = 65)	79.0 ±12.9 (n = 17)	74.0 ±17.9 (n = 231)
6–12 months post-surgery	5.5 ± 13.4 (n = 206)	7.3 ±16.3 (n = 51)	4.8 ±12.2 (n = 155)	4.3 ±10.6 (n = 152)	8.7 ±19.0 (n = 54)	3.7 ±10.6 (n = 15)	+5.6 ±13.6 (n = 191)
Sensitivity*		0.41		0.40		0.68	
p-value**	<0.001	<0.001	<0.001	<0.001	<0.001	<0.001	<0.001

VD–vaginal delivery; y.o.- years old

* 1-betta = 0.8; alfa = 0.05

** Wilcoxon signed-rank Test

**Fig 2:**
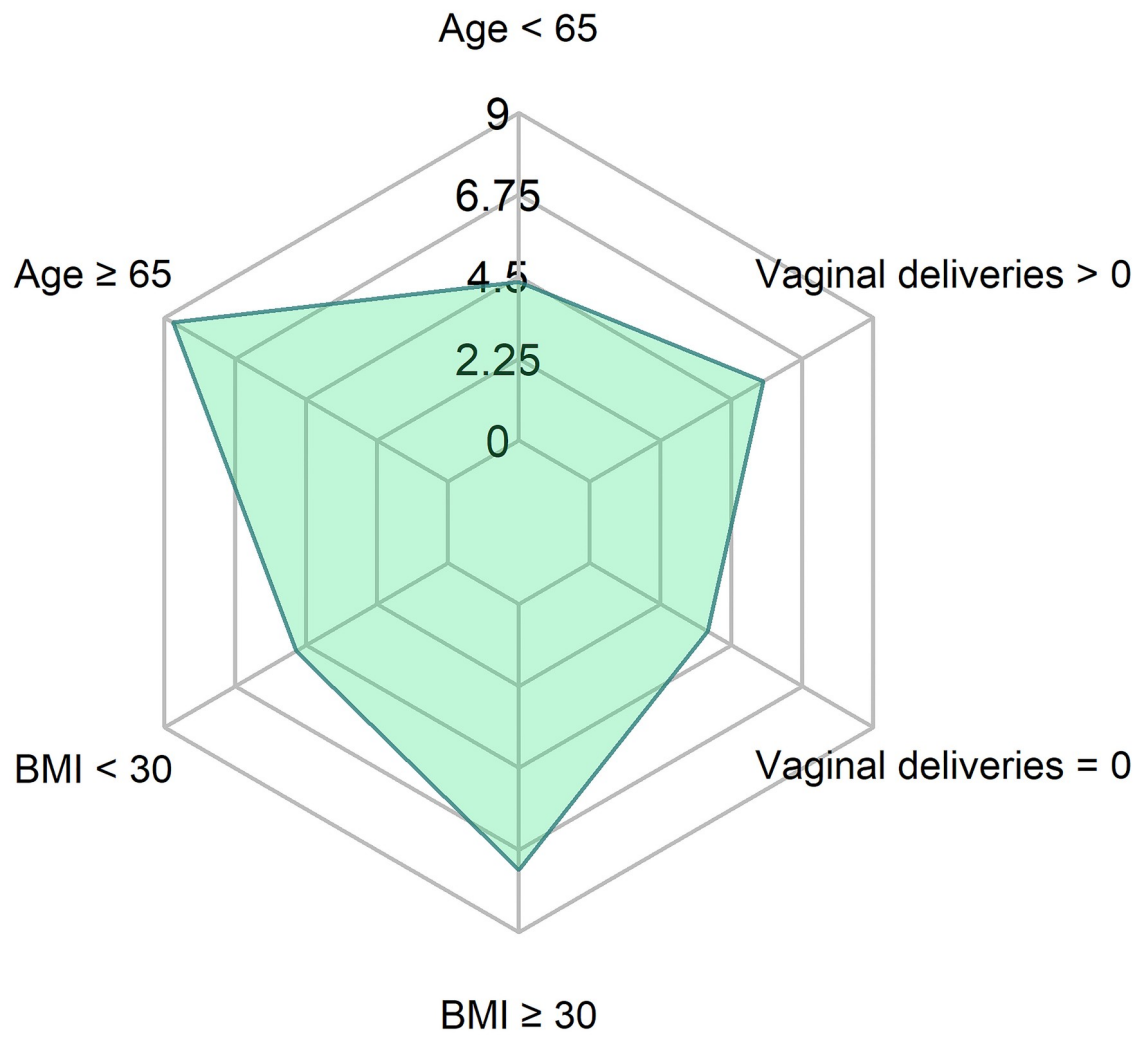
Mean improvement in IIQ-7 results 6–12 months after surgery. p-values: obese vs non-obese: 0.37; <65 y.o. vs ≥ 65 y.o.: 0.26; VD = 0 vs ≥ 1 VD: 0.36 (U Mann Whitney Test) IIQ-7—Incontinence Impact Questionnaire-7 VD–vaginal delivery BMI- body mass index (kg/m^2^).

No differences in subjective cure rates were observed between the groups depending on the demographic characteristics of patients is concerned (Fig 2).

In multivariate analysis, we did not observe any combinations of risk factors that might influence the objective and subjective cure rate.

## Discussion

The aim of the study was to evaluate whether commonly recognized risk factors of mid-urethral sling failure really have impact on the effectiveness of the retropubic MUS. The influence of obesity, age and parity were analyzed and no differences in the outcomes of the procedure were observed as compared to the control group.

Urinary incontinence is a world-wide problem that substantially influences the quality of life of the affected women. Although, as mentioned above, mid-urethral slings are the gold standard for SUI treatment with high cure rates and acceptable safety, there still remains a pending question concerning the causes of failure of the procedure.

Solving the questions regarding risk factors of sling failure is of great importance when it comes to counseling patients. As slings were being widely introduced into the market and are less invasive than other techniques, more patients with relative contraindications for long anesthesia (the elderly, the obese) received a chance for surgical treatment.

As far as the patient’s age is considered, there are conflicting data rating the effectiveness of the sling procedure. Toozs-Hobson analyzed 7600 cases and demonstrated that the global impression of improvement remained high in all age groups, but decreased slightly with age- more than 90% of women under 50 scored high effectiveness and the rate reduced to 70% in those over 80 years old [16]. The limitations of the abovementioned study included the follow-up rate reaching only 54% and only subjective rating of the effectiveness. Sharp et al., in a review of both retrospective and prospective studies, demonstrated that surgery for SUI in older women improves symptoms, but not to the extent seen in younger women [17]. In another study, concerning the cumulative risk of repeated SUI surgery, it was shown that the 5-year risk of undergoing a repeat SUI surgery was less than 10% with higher risks for women 65 years or older [18]. In a survey study including patients who underwent retropubic sling procedure it was demonstrated that the older group had a significantly lower success rate compared to younger patients (53.1% vs 83.6%) [19].

All these data resulted in the observation that despite the fact that mid-urethral slings are truly minimally invasive, elderly women do not seem to benefit, probably because of doubts connected with effectiveness of the procedure [20]. Meanwhile, in another cohort retrospective study where 488 patients over 70 years old were included, the authors showed that there are no differences in sling success rates between the elderly and a younger population [21]. Our results are in line with the last cited study. We did not show any differences between the group of younger and elderly patients as far as subjective and objective cure rate is considered. When discussing the results cited above, it should be noted that in most of the studies, the success rates were evaluated based on surveys and the follow up rate was relatively low. Therefore, our study might be perceived as more reliable due to the fact that in all cases evaluation was performed on the basis of objective and subjective criteria during follow up visits and the follow-up rate was significantly higher than in the cited papers. On the other hand, a relatively short period of observation constitutes the limitation of the present study. Nevertheless, taking into consideration high follow-up rate and both subjective and objective evaluations (both cough and 1-hour pad test as well as incontinence impact questionnaire), they seem to be more reliable than survey studies.

Another widely discussed issue concerning the effectiveness of sling procedures is obesity and overweight. In a 5-year follow-up of 136 women who underwent retropubic sling procedures, the authors showed significantly worse subjective and objective results in obese patients as compared to other subjects (98 vs 71%, respectively, p = 0.004) [22].

In a randomized controlled trial including 12 months’ follow-up in obese patients, significantly worse results were shown in this group of patients. Objective cure rates differed, with 85.6% of non-obese women vs 67.8% of obese women. Subjective cure rate was 85.8% for non-obese women vs 70.7% for obese women [23]. The same group of authors confirmed that in a longer follow-up period (5 years), obesity sill had an impact on the cure rate [8].

Berger at al. showed that obese women undergoing retropubic TVT surgery had a 3.56-fold increased odds of short-term complaints of SUI compared with normal weight patients, with the limitation of short term observations (2 weeks and 2 months only) [24]. Similar results were obtained in a retrospective cohort study analyzing single incision slings (SIS). The authors reported a trend towards lower objective efficacy of SIS with increasing body weight, with a significant difference between obese women and normal subjects: 75% vs 91.3%, p = 0.049; OR 3.74 (95% CI 1.19–11.76) [25].

On the other hand, there have been reports demonstrating that in a retrospective 12 months’ evaluation, objective cure rates in the normal and overweight groups did not differ and were 96.2% and 94%, respectively (p = 0.47) [26].

In the present study, we demonstrated equal effectiveness and cure rates between obese and non-obese patients in a 12-month observation. Moreover, we did not observe any negative influence of the combination of obesity and age on the effectiveness of the procedure. Our results contradict those of Hellberg et al., who reported that these factors were connected with worse sling outcome [27]. The method of obtaining data is probably one of the reasons why the results of this study contradict ours’. In the abovementioned analysis, the effectiveness was assessed with the use of a mailed questionnaire and the authors had no chance to find out about various factors influencing the patients’ reports such as concomitant complaints (i.e. overactive bladder syndrome, pain syndrome, lower urinary tract infections).

We assume that the main factor responsible for the worse outcome in obese patients in the abovementioned studies might be the problem of proper sling location. In our previous study, we showed that inappropriate sling location beneath proximal part of urethra is connected with persistent or recurrent SUI [9]. In another observation, we demonstrated that obese patients have significantly longer urethral length as compared to non-obese, and thus sling procedure is of higher risk of failure because of suboptimal sling location when using standard surgery technique [28]. In the present study we measured urethral length in all cases individually and the surgery technique was modified accordingly so that the vaginal incision was made in 1/3 of the urethral length, obtaining optimal sling location in all cases. The bias connected with suboptimal sling location was therefore ruled out.

Taking into consideration our observations based on the objective and subjective cure rate with adequate sample size and power of the analysis as well as having confidence of proper sling location, we postulate that there is no need to delay the surgery because of obesity, independently of the general health benefits form weight reduction.

The last analyzed risk factor for lower effectiveness of sling procedure was parity. It is well recognized that parity is connected with higher incidence of SUI and hypermobility of the bladder neck. It has been shown that patients with a history of lower number of pregnancies and lower number of deliveries had significantly higher operation success rates [29]. In the current study, we demonstrated that the number of vaginal deliveries had no influence on the sling procedure effectiveness. It should be stressed that in this case we also ruled out the main cause of sling ineffectiveness, i.e. in a form of inappropriate sling location. This seems to be of a great importance, since we previously showed that parity is connected with shorter urethras and at the same time with higher risk of sling failure [28].

The differences between our results and the studies cited previously may be connected both with the different surgery technique (we individualized the sling location according to the urethral length) and with the clearly defined study group with exclusion of cases of previous POP repair in our study as well as with the exclusion of cases with mixed urinary incontinence as well as other voiding problems.

The main limitation of the presented study is a short observational period (up to 12 months). The effectiveness of the procedure needs to be evaluated in longer observation. On the other hand, this is the first evaluation of combined risk factors influence on the procedure effectiveness most authors considered one isolated demographic feature. Moreover, the prospective character of the study as well as the fact that both objective and subjective cure rates were analyzed guarantee a proper quality of the presented results.

## Conclusions

In the present study, no differences between objective and subjective cure rates were shown in relation to BMI, age and parity of patients who underwent mid-urethral sling procedure in 12 months observation. Moreover, we did not demonstrate any combination of the abovementioned features as risk factors for mid-urethral sling failure. We therefore postulate that demographic features do not influence sling effectiveness and that probably the most important factor playing a role is the surgery technique as well as proper selection of patients as far as the diagnosis is concerned.
